# Duration of pretomanid/moxifloxacin/pyrazinamide therapy compared with standard therapy based on time-to-extinction mathematics

**DOI:** 10.1093/jac/dkz460

**Published:** 2019-11-12

**Authors:** Shashikant Srivastava, Devyani Deshpande, Gesham Magombedze, Johanna van Zyl, Kayle Cirrincione, Katherine Martin, Paula Bendet, Alexander Berg, Debra Hanna, Klaus Romero, Dave Hermann, Tawanda Gumbo

**Affiliations:** 1 Center for Infectious Diseases Research and Experimental Therapeutics, Baylor Research Institute, Baylor University Medical Center, Dallas, TX, USA; 2 Praedicare Laboratories, Dallas, TX, USA; 3 Critical Path to TB Drug Regimens, Critical Path Institute, Tucson, AZ, USA; 4 Bill & Melinda Gates Foundation, Seattle, WA, USA; 5 Lung Infection and Immunity Unit, Division of Pulmonology and UCT Lung Institute, Department of Medicine, University of Cape Town, Cape Town, South Africa

## Abstract

**Objectives:**

Animal models have suggested that the combination of pretomanid with pyrazinamide and moxifloxacin (PaMZ) may shorten TB therapy duration to 3–4 months. Here, we tested that in the hollow-fibre system model of TB (HFS-TB).

**Methods:**

A series of HFS-TB experiments were performed to compare the kill rates of the PaMZ regimen with the standard three-drug combination therapy. HFS-TB experiments were performed with bacilli in log-phase growth treated for 28 days, intracellular bacilli treated daily for 28 days and semi-dormant *Mycobacterium tuberculosis* treated with daily therapy for 56 days for sterilizing effect. Next, time-to-extinction equations were employed, followed by morphism transformation and Latin hypercube sampling, to determine the proportion of patients who achieved a time to extinction of 3, 4 or 6 months with each regimen.

**Results:**

Using linear regression, the HFS-TB sterilizing effect rates of the PaMZ regimen versus the standard-therapy regimen during the 56 days were 0.18 (95% credible interval=0.13–0.23) versus 0.15 (95% credible interval=0.08–0.21) log_10_ cfu/mL/day, compared with 0.16 (95% credible interval=0.13–0.18) versus 0.11 (95% credible interval=0.09–0.13) log_10_ cfu/mL/day in the Phase II clinical trial, respectively. Using time-to-extinction and Latin hypercube sampling modelling, the expected percentages of patients in which the PaMZ regimen would achieve sterilization were 40.37% (95% credible interval=39.1–41.34) and 72.30% (95% credible interval=71.41–73.17) at 3 and 4 months duration of therapy, respectively, versus 93.67% (95% credible interval=93.18–94.13) at 6 months for standard therapy.

**Conclusions:**

The kill rates of the PaMZ regimen were predicted to be insufficient to achieve cure in less than 6 months in most patients.

## Introduction 

The international community has been developing regimens to shorten therapy from the current 6 months to 2–4 months for drug-susceptible TB and from 12–14 months to 6 months for MDR-TB. To achieve this, new compounds are being developed and used in novel combination regimens comprising old and new drugs. The nitroimidazole-oxazine compound pretomanid, which is a nitric oxide donor that interferes with the *Mycobacterium tuberculosis* respiratory system to kill both aerobic and anaerobic bacilli, is one such candidate.^1–3^ The bactericidal and sterilizing activities of pretomanid were confirmed in mouse studies; results suggested that when combined with pyrazinamide, the pretomanid/pyrazinamide combination could lead to more rapid killing of *M. tuberculosis*.^4^ Next, the pretomanid/pyrazinamide backbone was combined with moxifloxacin to create the PaMZ combination regimen, which showed a faster sterilizing effect than the first-line drugs in the guinea pig model of TB.^5^^,^^6^ These results suggested that the PaMZ regimen should be tested as a regimen to shorten therapy duration in patients.

In clinical trials, PaMZ regimens showed a sustained microbial kill rate over 8 weeks of therapy; the kill rates of PaMZ (with 200 mg/day pretomanid) based on linear regression were 0.16 (95% credible interval=0.13–0.18) log_10_ cfu/mL/day versus 0.11 (95% credible interval=0.09–0.13) log_10_ cfu/mL/day with standard-dose isoniazid, rifampicin, pyrazinamide and ethambutol.^7^ This was followed by the clinical trial ‘Shortening Treatment Regimen by Advancing Novel Drugs’ (STAND), meant to test PaMZ for potential shortening of the duration of therapy, following the patients for up to 24 months.^8^ However, based on results from the Phase IIb NC-005 trial, it appeared that a combination of bedaquiline, pretomanid, moxifloxacin and pyrazinamide could be a better alternative compared with the PaMZ regimen. Therefore, the STAND trial was terminated early.^8^ Here, the hollow-fibre model system of TB (HFS-TB) was used to identify kill rates of the PaMZ regimen versus the standard first-line combination therapy regimen to investigate whether STAND would have succeeded in shortening therapy duration. Results were analysed using a *de novo* model that traces the trajectories of different *M. tuberculosis* subpopulations to extinction in the HFS-TB, which are then mapped to extinction of these bacterial subpopulations in patients using morphism mapping and transformations.^9^ Morphism transformation followed by Latin hypercube sampling (1000 patient simulations of time-to-extinction distributions, given different bacterial kill slopes and bacterial burden) were then applied to predict clinical outcomes of each regimen for different therapy durations.^9^ The study, analyses and manuscript were completed in September 2017, prior to release of STAND trial results.

## Materials and methods

### Bacterial strains, cells, drugs and other supplies

*M. tuberculosis* laboratory strains H37Ra (ATCC 25177) and H37Rv (ATCC 27294) were used in the HFS-TB experiments. Culture conditions for stock bacterial strains and the human-derived THP-1 cells (ATCC TIB-202) were the same as reported in our previous publications.^10–12^ Study drugs were purchased from Sigma−Aldrich (St Louis, MO, USA), Baylor Medical Center campus pharmacy and BOC Sciences (NY, USA). The mycobacterial growth indicator tube (MGIT) system and related supplies were purchased from Becton Dickinson (Franklin Lakes, NJ, USA).

### Hollow fibre studies

Detailed descriptions of the HFS-TB for bactericidal and sterilizing activity, as well as that for the intracellular *M. tuberculosis* model, have been previously published.^11–13^ Briefly, 20 mL of either log-phase growth or semi-dormant *M. tuberculosis* at pH 5.8 or *M. tuberculosis*-infected THP-1 monocytes was inoculated into the peripheral compartment of each HFS-TB.^11–13^ Drugs were infused into the central compartment of the HFS-TB to achieve human concentration–time profiles of each drug in the combination regimen. For the PaMZ regimen, concentration–time profiles mimicked were for moxifloxacin AUC_0–24_ of 52 mg·h/L (equivalent to 400 mg) and pyrazinamide AUC_0–24_ of 656 mg·h/L (equivalent to 2 g), administered daily, at a half-life of 12 h for each drug. For pretomanid, reported to have an elimination half-life of 17.2–24.6 h in patients with TB, and a mean half-life of 18 h in another study, concentration–time profiles mimicked were for 200 mg of pretomanid at a half-life of 18 h.^1^^,^^14^ For the standard-therapy regimen, the concentration–time profiles were for rifampicin 600 mg (AUC_0–24_ = 28 mg·h/L), isoniazid 300 mg (AUC_0–24_ = 33 mg·h/L) and pyrazinamide 2 g, administered daily, to achieve a half-life of 3 h for rifampicin and isoniazid, and 12 h for pyrazinamide. Three untreated HFS-TB units served as negative controls.

Three separate HFS-TB studies were performed to evaluate: (i) bactericidal effect; (ii) intracellular infection; and (iii) sterilizing effect of the PaMZ regimen and the standard regimen. For bactericidal effect, *M. tuberculosis* H37Rv in log-phase growth was inoculated into the peripheral compartment of each HFS-TB unit and treatment started 24 h later that continued daily for 28 days. For the intracellular infection model, THP-1 monocytes infected with *M. tuberculosis* H37Ra were inoculated into the peripheral compartment and treated daily with each of the regimens for 28 days. For the semi-dormant *M. tuberculosis*, H37Rv that had been maintained at pH 5.8 for 4 days was inoculated into HFS-TB, which was then treated with each of the regimens for 56 days. In each experiment, the concentration–time profile of each drug achieved in each HFS-TB was validated by sampling the central compartments at 1, 6, 10, 18, 21, 23.5, 25, 30, 34, 42, 45 and 47.5 h after the first dose. The peripheral compartments were sampled at predetermined timepoints, including every 7 days, and samples were processed for estimation of bacterial burden using both cfu counts and time to positivity (TTP) from the MGIT, as previously described.^10–13^ To identify drug-resistant subpopulations, the samples were also cultured on agar supplemented with three times the MIC of each drug in the regimen and incubated at 37°C under 5% CO_2_ for 5 weeks before the colonies were counted.

### Drug concentration assays

A multiplexed LC-MS/MS method was used for all drug concentration measurements. The methods used to measure drug concentrations of isoniazid, pyrazinamide, rifampicin and moxifloxacin were published previously and were used without any modification.^15–18^ For pretomanid, an LC-MS/MS assay was developed where calibrator, controls and internal standards were included in each analytical run for quantitation. The stock solutions of the standards and internal standard (metronidazole) were prepared in 80:20 methanol/water at a concentration of 1 mg/mL and stored at −20°C. A seven-point calibration curve (range 0.05 to 10 mg/L) was prepared by diluting the stock solution in drug-free Middlebrook 7H9 broth. Quality control samples were prepared by spiking Middlebrook 7H9 broth with stock standards for two levels of controls. Samples were prepared in 96-well microtitre plates by the addition of 10 μL of calibrator, quality controls or sample to 190 μL of 0.1% formic acid in water containing 1 μg/mL of each of the internal standards, followed by vortexing then centrifuging at 4000 rpm for 5 min to remove any cellular debris. Chromatographic separation was achieved by injecting 2 μL on an Acquity UPLC HSS T3 1.8 μm 50 × 2.1 mm analytical column (Waters) maintained at 30°C at a flow of 0.2 mL/min with a binary gradient and a total run time of 6 min. Solvents for UPLC were: A, water containing 0.1% formic acid; and B, methanol containing 0.1% formic acid. The initial gradient condition was 5% B for the first 1.5 min and was increased in a linear fashion to 100% B at 1.8 min and held constant for 1.5 min. At 4.5 min, the flow was reset to initial conditions for 1.5 min. The flow from the column was delivered to the Z-spray source from the period of 0 to 3.25 min; the compounds were detected by MS/MS using positive electrospray ionization for all compounds. The capillary voltage was set to 3.5 kV with a desolvation temperature and gas flow of 400°C and 800 L/h, respectively. The cone gas (nitrogen) flow was set at 100 L/h with a collision gas (argon) flow of 0.21 mL/min. Sample injection and separation was performed by an Acquity UPLC interfaced with a Xevo TQ mass spectrometer (Waters). All data were collected using MassLynx version 4.1 SCN810. The between-day percentage coefficient of variation (%CV) for analysis of low and high controls [low (high) controls] of pretomanid was 12% (11%). The intraday %CV for low and high controls was 10% (6%). The lower limit of detection of this method was 0.05 mg/L.

### Pharmacokinetic analysis

Drug pharmacokinetics in the HFS-TB were modelled using a one-compartment model with first-order input and elimination, using ADAPT 5.^15^ The observed peak concentration (*C*_max_) and area under the curve (AUC_0–24_) were used to calculate the peak concentration to MIC (*C*_max_/MIC) ratio, AUC_0–24_/MIC ratio and the percentage of time that concentration persists above the MIC (%*T*_>MIC_) for each drug in the regimen.

### Pharmacodynamic analysis

To allow for comparisons of the kill rates in the HFS-TB with clinical work done with the PaMZ regimen already, we calculated kill rates using linear regression.^1^^,^^7^ In this model, the slope of log_10_ bacterial population versus time is assumed to be linear. However, it is well established that bacillary sputum decline rates in patients are not linear and nor are decline rates and trajectories of bacterial subpopulations in the HFS-TB.^19^ As an alternative analysis method, we have developed a model that tracks the trajectories of *M. tuberculosis* populations for bactericidal effect, sterilizing effect and intracellular bacteria from start of treatment to extinction of the population, in both the HFS-TB and in patients on combination therapy.^9^ The model-based HFS-TB trajectories were mapped to those in patients, allowing translation of time to extinction of bacterial subpopulations. Thus, the intrinsic bacterial growth rates were estimated, as were the drug kill rates for each specific regimen, based on the model. *In silico* simulations using Latin hypercube sampling were then applied to generate parameter samples representing 1000 HFS-TB treatment outcomes for the PaMZ regimen and the standard regimen. The bacterial time to extinction computed for each outcome in the HFS-TB was then translated to patients using the mapping transformations, thereby predicting the time taken to eliminate all bacterial subpopulations in patients treated with each regimen.^9^ When this method was tested in the past, it correctly identified cure rates of 6 months and 4 months duration experimental and standard regimens in clinical trials.^9^

## Results

**Figure 1.**
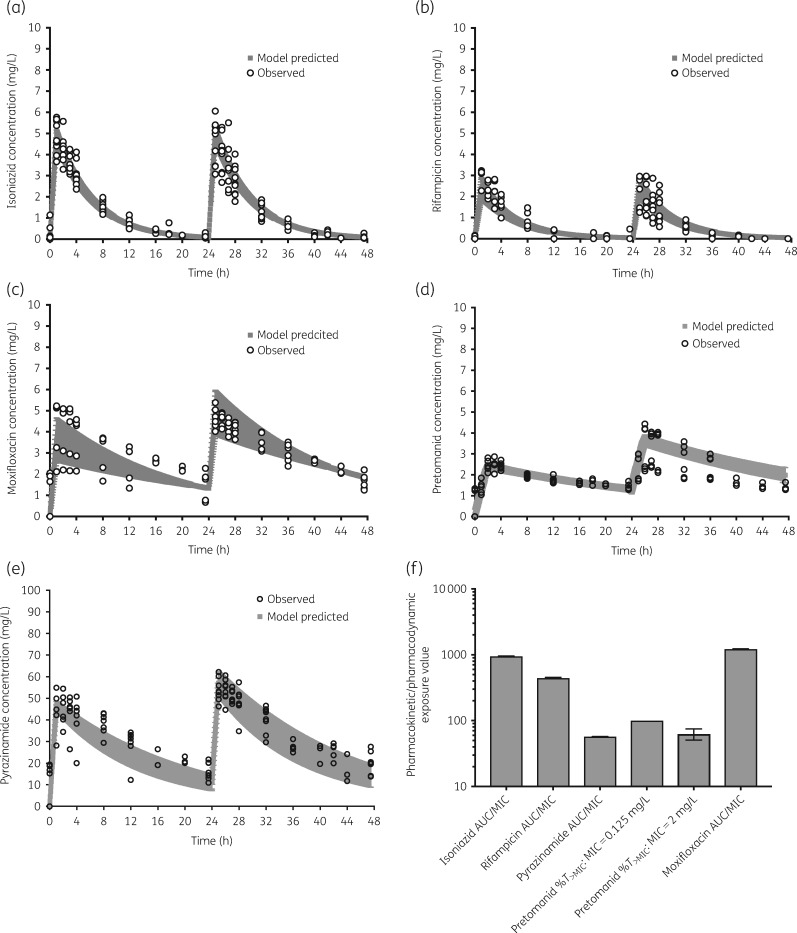
Concentration–time profiles of the drugs. (a–e) Pharmacokinetic model-derived concentration–time profiles based on drug concentrations measured in the HFS-TB. Open symbols are observed concentrations and shaded areas are 95% confidence bounds. (f) Pharmacokinetic/pharmacodynamic exposure achieved for each drug with errors bars that are 95% credible intervals. Error bars are very narrow for each drug, except for pretomanid (%*T*_>MIC_: MIC=2 mg/L), which shows modest error bars.

The MICs of isoniazid, rifampicin, pyrazinamide and moxifloxacin for the laboratory strains in log-phase growth are shown in Table 1. For pretomanid we also examined the MIC at pH 5.8 (under semi-dormant conditions) and for intracellular *M. tuberculosis*, as shown in Table 1. Concentration–time profiles across all HFS-TB replicates within a given experimental condition for each drug were pooled for pharmacokinetic modelling, with results shown in Figure 1(a–e). The pharmacokinetic/pharmacodynamic exposures identified for each drug, based on the pharmacokinetic/pharmacodynamic index that drives the particular drug’s efficacy, are shown in Figure 1(f) and reflect those achieved in TB cavities.^20–25^ The elimination rate constants (%CV between replicates) were 0.18 (8.58%) for isoniazid, 0.19 (16.51%) for rifampicin, 0.06 (2.0%) for pyrazinamide, 0.06 (10.90%) for moxifloxacin and 0.04 (6.99%) for pretomanid. The %CV values demonstrate little to no technical variation between replicates and between experiments, and are below our recommended 20% cut-off point for acceptable technical variation.

**Table 1.** MICs (mg/L) for *M. tuberculosis* laboratory strains used in the study

	H37Ra	H37Rv
Isoniazid	0.06	0.06
Rifampicin	0.06	0.06
Pyrazinamide	25	25
Moxifloxacin	0.125	0.125
Pretomanid	0.125, 0.125^a^, 2.0^b^	0.125, 2.0^b^

a Intracellular MIC.

b MIC at acidic pH of 5.8.

**Figure 2.**
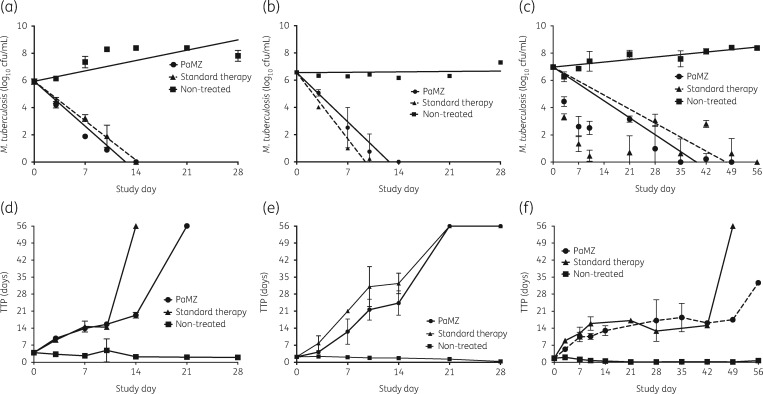
Kill curves and TTP for standard and PaMZ regimens. As shown in (a), (b) and (c), there was no difference in the kill curves of the standard regimen versus the PaMZ regimen against intracellular, log-phase growth and semi-dormant *M. tuberculosis*, respectively. Both regimens took virtually the same time to sterilize the HFS-TB. However, by TTP, the PaMZ regimen took longer to kill the intracellular *M. tuberculosis* compared with the standard regimen (d), was similar to the standard therapy against the log-phase growth *M. tuberculosis* (e) and failed to sterilize the systems in the experiment with the semi-dormant *M. tuberculosis* (f). Thus, in the HFS-TB, PaMZ was not superior to the current standard regimen.

Figure 2 shows the time–kill curves of the regimens for intracellular bacteria (Figure 2a), log-phase growth extracellular bacteria (Figure 2b) and sterilizing effect (Figure 2c), based on log_10_ cfu/mL count. TTP is more sensitive than solid agar cfu/mL counts for HFS-TB experiments as observed for clinical trials^23–29^ and the corresponding TTP for each treatment regimen is shown in Figure 2(d–f). Based on TTP, the PaMZ regimen took longer to eliminate the intracellular and semi-dormant *M. tuberculosis* compared with standard therapy (Figure 2d and f, respectively). In contrast, application of linear regression (shown in Table 2) revealed slopes that were similar for both PaMZ and standard therapy. The slopes based on the 56 day experiment for sterilizing effect were very similar to the 56 day-based linear regression rates of 0.16 (95% credible interval=0.13–0.18) log_10_ cfu/mL/day for PaMZ and 0.11 (95% credible interval=0.09–0.13) log_10_ cfu/mL/day for standard therapy, in the Phase II clinical trial.

**Table 2.** Kill slopes for regimens based on linear regression

	PaMZ	Standard therapy	Non-treated control
Intracellular	0.47 (0.55–0.39)	0.41(0.44–0.39)	−0.07 (−0.01 to −0.16)
Log-phase	0.52 (0.59–0.44)	0.57 (0.74–0.39)	−0.02 (−0.01 to −0.05)
Semi-dormant	0.18 (0.23–0.13)	0.15 (0.21–0.08)	−0.02 (−0.02 to −0.03)

The kill rates are shown as log_10_ cfu/mL/day and the numbers in parentheses are the 95% credible intervals.

**Figure 3.**
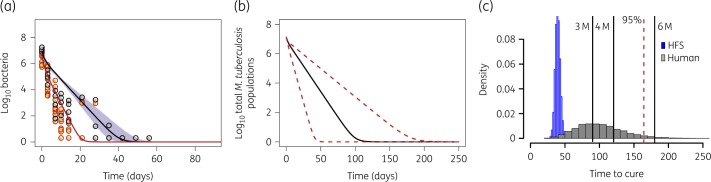
Time-to-extinction in HFS-TB and morphism-based translation to patients on the standard regimen. Standard regimen kill curve for both bacteria in log-phase growth plus intracellular and in the semi-dormant state. (a) The model explains the depletion of both subpopulations; the orange circles show log-phase/intracellular *M. tuberculosis* observations, while the grey circles show semi-dormant population observations in the HFS-TB, based on both TTP and log_10_ cfu/mL. The lines are the model fitting, with the red line representing extracellular log-phase growth and intracellular *M. tuberculosis*, while the black line is for the persister population. (b) The HFS-TB data were then transformed based on structure-preserving mapping (morphism) to kill curves in patients; shown is the decline of the combined bacteria subpopulations (log-phase plus intracellular plus semi-dormant summation) in patients with TB. Slower depletion of persisters is demonstrated after the transformation, with a wide spread of time-to-extinction. (c) Distribution of time-to-extinction after Latin hypercube sampling. The time-to-extinction is the minimum duration of therapy needed with that regimen. For the standard therapy regimen shown here 6 months therapy would result in time-to-extinction in about 94% of patients, if doses were optimized. 3 M, 3 months; 4 M, 4 months; 6 M, 6 months. This figure appears in colour in the online version of *JAC* and in black and white in the print version of *JAC*.

**Figure 4.**
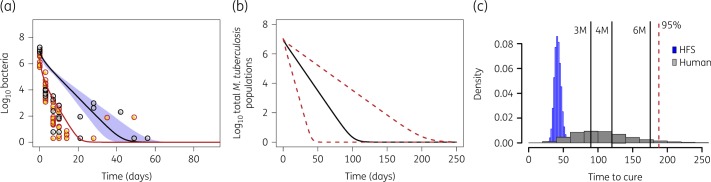
Time to extinction on the PaMZ regimen. Observed HFS-TB model results versus patient-predicted outcomes for the PaMZ regimen. (a) Intracellular and extracellular bacteria in log-phase growth are represented by orange circles and bacteria in the semi-dormant state are represented by grey circles. The separate bacterial subpopulations are explained with the red and black model fitted lines, respectively. (b) The HFS-model data-constrained trajectories are then translated to show the patient-associated bacteria–time kinetics during therapy with the PaMZ regimen. (c) The proportion of patients that will get cured (i.e. achieve bacterial extinction) is low with 3 months or 4 months duration of therapy, and would be virtually identical to the standard regimen if administered for 6 months duration. 3 M, 3 months; 4 M, 4 months; 6 M, 6 months. This figure appears in colour in the online version of *JAC* and in black and white in the print version of *JAC*.

As seen in the time–kill curves presented in Figure 2, the kill slopes for both regimens are not linear. Therefore, time-to-extinction modelling was applied whereby cfu/mL counts and TTP are shown in Figures 3(a) and 4(a), respectively. Figures 3(b) and 4(b) show the same trajectories, now transformed to patients’ lungs/sputum, based on a transformation factor we derived using HFS-TB data and two clinical trials in the past.^9^ When *M. tuberculosis* populations have reached extinction, the patient has been cured of the infection (though they may continue suffering consequences of the infection). Hence, the patient-specific time to extinction indicates a minimum duration of therapy for cure and the distribution of time-to-extinction estimates (Figures 3c and 4c) can be used to calculate the duration required to cure a specific portion of the population.^9^ Inspection of Figures 3 and 4 shows that the slopes (i.e. time-to-extinction trajectories) for PaMZ were virtually identical to those of standard therapy, as was the distribution of patient-specific times to extinction following translation of HFS-TB results to predict clinical outcome. Hence, PaMZ did not provide improved kill rates relative to the standard regimen; the kill rates of PaMZ are therefore insufficient to shorten the treatment duration from 6 months to 4 months. This is apparent in the model-based ‘success’ rates for standard therapy and PaMZ at selected treatment durations, as shown in Table 3.

**Table 3.** Percentage (95% credible interval) of patients with bacterial populations reaching extinction

	Therapy duration, months		
	3	4	6
PaMZ	40.37 (39.41–41.34)	72.3 (71.41–73.17)	97.47 (97.14–97.76)
Standard therapy	38.25 (37.30–39.21)	66.34 (65.41–67.26)	93.67 (93.18–94.13)

## Discussion

Identification of optimal duration of therapy is a limitation of preclinical models of TB as they are models of infection, but not of human disease. Identifying the attributes of the model that can be mapped to patients and then determine the magnitude, shape and direction of the response vector is important in understanding translation.^9^ Such a mapping approach, coupled with extinction mathematics, allows for the determination of the upper limits of duration of therapy that could cure TB. We applied this approach to the PaMZ regimen in the HFS-TB, as recently examined in the STAND trial, and found that a treatment duration of 3–4 months would have resulted in large proportions of patients not reaching extinction of the *M. tuberculosis* subpopulation. While not all patients who have not achieved extinction will fail therapy, all patients who fail therapy or relapse will, by definition, not have achieved bacterial population extinction. Thus, failure rates would be dictated by the proportion of patients who failed to sterilize all *M. tuberculosis.*

Second, the model-derived slopes give a target for newer regimens derived from the PaMZ regimen. Given that sterilization is slower than the bactericidal effect, the former is the rate-limiting process for shortening of treatment duration. The data suggest that the slopes for regimens that would shorten therapy would need to be considerably steeper, at least 33% steeper than those for standard therapy. In order to get to the destination sooner, the speed would need to be increased proportionately; if a 2 month regimen is required then the sterilizing effect slopes would need to be three times faster than for standard therapy. The focus should therefore be on types of clinical studies that are designed to examine the sterilizing effect rates rather than the bactericidal effect rates. However, bactericidal effect also plays an important role in limiting the infectiousness of patients and has an important public health role. The combination of a preclinical drug development tool that has been mapped to patient response, such as HFS-TB, and the mathematical modelling could be used to predict the therapy duration with the new combination in development to make a go/no-go decision, followed by clinical trials focused on defining sterilizing effect slopes in a minimum number of patients based on the variance of time to extinction, followed by a larger Phase III study.^30–34^

Our study has the limitation of using only the standard laboratory strains. However, when we used these strains in the past and performed the simulations to predict the clinical outcomes in patients with TB, the predictive accuracy was over 94%.^33^ Moreover, we performed our initial mapping results using these very same strains versus clinical responses, which were then validated.^9^ Second, all models have limitations and what we present are predictions; the final proof is always with clinical trials. Thus, we await release of the clinical results of the STAND trial, even though the study was stopped before completion. Third, other TB infection models such as animal models can be used, provided the treatment response in the model is mapped to that in patients. Thus, the HFS-TB is not the sole preclinical model to be used. However, HFS-TB has the advantage of repetitive sampling to create a set of TTP values from the same unit, much like sampling each patient’s sputum, so that the two sets are easy to compare and translate using structure-preserving mapping.

## Funding

This work was supported by a grant from the Critical Path to TB Regimens (CPTR) of the Critical Path Institute (grant number OPP1031105 to T.G.). 

## Transparency declarations

T. Gumbo founded Praedicare LLC. All other authors: none to declare. 

### Author contributions

D. Hanna, D. Hermman and T. Gumbo designed the study. S. Srivastava and T. Gumbo supervised the study. S. Srivastava, D. Deshpande, J. van Zyl, K. Cirrincione, K. Martin and P. Bendet performed the hollow fibre experiments. G. Magombedze performed the time-to-extinction modelling. A. Berg and T. Gumbo performed the pharmacokinetic modelling. T. Gumbo performed the pharmacokinetic/pharmacodynamic modelling. S. Srivastava and T. Gumbo wrote the draft manuscript followed by extensive comments and rewriting from A. Berg, D. Hanna and K. Romero. All authors read and approved the final version of the manuscript.
